# Significance of Glutathione Peroxidase 1 and Caudal-Related Homeodomain Transcription Factor in Human Gastric Adenocarcinoma

**DOI:** 10.1155/2013/380193

**Published:** 2013-10-21

**Authors:** Jing Jing Han, De Rong Xie, Li Li Wang, Ye Qing Liu, Gong Fa Wu, Qing Sun, Yan Xian Chen, Ying Wei, Zhi Quan Huang, Hai Gang Li

**Affiliations:** ^1^Department of Pathology, Zengcheng People's Hospital, Zengcheng City 511300, China; ^2^Department of Pathology, Sun Yat-Sen Memorial Hospital, Key Laboratory of Malignant Tumor Gene Regulation and Target Therapy of Guangdong Higher Education Institutes, Sun Yat-Sen University, Guangzhou 510120, China; ^3^Department of Oncology, Sun Yat-Sen Memorial Hospital, Key Laboratory of Malignant Tumor Gene Regulation and Target Therapy of Guangdong Higher Education Institutes, Sun Yat-Sen University, Guangzhou 510120, China; ^4^Department of Oral and Maxillofacial Surgery, Sun Yat-Sen Memorial Hospital, Key Laboratory of Malignant Tumor Gene Regulation and Target Therapy of Guangdong Higher Education Institutes, Sun Yat-Sen University, Guangzhou 510120, China

## Abstract

*Aim*. To investigate the expressions of glutathione peroxidase 1 (GPX1) and caudal-related homeodomain transcription factor (CDX2) in GAC and their correlation with clinicopathological features and tumor cell proliferation. *Methods*. The expressions of GPX1, CDX2, and Ki67 were immunohistochemically evaluated in 172 GAC specimens. The association of GPX1 and CDX2 with patient's clinicopathological features and Ki67 positive rate was analyzed statistically. *Results*. In 172 cases of GAC, the expression of GPX1 was weaker than that in adjacent normal mucosa, and the expression of CDX2 was higher than that in adjacent normal mucosa. High expression GPX1 strong-expression was associated with differentiation, Lauren type, WHO type and extensive lymph node metastasis of GAC. High expression of CDX2 was associated with differentiation, Lauren type, WHO type, extensive lymph node metastasis, and TNM of GAC. Survival curves showed that expressions of GPX1 and CDX2 were factors of good outcome (*P* = .03 and .02, resp.). According to multivariate analysis, only lymph node metastasis, TNM stage, and CDX2 expression were independently associated with survival. In addition, a strong association of GPX1 expression was noted with Ki67 and CDX2. *Conclusions*. The expression of GPX1 and CDX2 may play a role in the carcinogenesis, differentiation, and progression of GAC, and CDX2 may be an independent prognostic factor.

## 1. Introduction

Gastric adenocarcinoma (GAC) is one of the most common fatal malignancies in the world. The incidence varies considerably between geographical areas, with a higher incidence in China and other Asian countries than in Western Europe and the United States [1]. Stomach cancer was the fourth most common cause of cancer-related death from cancer in Europe in 2012 [2]. In China, gastric cancer was the second most common cancer in 2009 [3]. Patients with gastric cancer that is limited to the mucosa and submucosa have an excellent prognosis, with a 5-year survival rate of over 90% after surgery [4]. In contrast, the prognosis for patients with advanced cancer is generally poorer and less predictable. At present, therapeutic decisions are based on clinicopathological parameters, including age, tumor node metastasis (TNM) stage, and histological grade. Although useful, these factors often fail to differentiate more aggressive tumor types from less aggressive tumor types [5]. As GAC is a markedly heterogeneous disease with respect to histological features and biological characteristics especially in the advanced stages, previous studies have shown that its biological behavior and prognosis could be significantly different among the patients with the same stage, histological type, or differentiation grade. Searching for special markers that are closely related to bionomic characteristics and outcome is still one of the major foci of research on GAC, although a number of biomarkers have already been found to be involved in the development and progression of GAC. 

The antioxidant enzyme glutathione peroxidase 1 (GPX1) is part of the enzymatic antioxidant defense in normal cells. GPX1 catalyses the reduction of hydrogen peroxide, organic hydroperoxide, and lipid peroxides by reduced glutathione, thereby protecting cells against oxidative damage [6–8]. Decreased activity of these antioxidant enzymes may increase oxidative stress and damage to several biomolecules, including DNA, which may initiate or promote neoplastic transformation in affected tissues [9]. The loss of GPX1 expression was associated with aggressiveness and poor survival in patients with gastric cancer [10].

The homeobox transcription factor caudal-related homeodomain transcription factor (CDX2) plays a crucial role in intestinal cell fate specification, and it is a critical determinant of intestinal homeostasis both during development and throughout adult life. Furthermore, there are substantial pieces of evidence supporting the crucial role of CDX2 in carcinogenesis of the digestive tract. CDX2 was shown to inhibit cell growth and migration *in vitro*, as well as the dissemination of colon tumor cells *in vivo* [11]. CDX2 reduction increases the progression of chemically induced colorectal cancers [12]. Conversely, under certain pathological conditions, CDX2 becomes abnormally expressed in other organs of the digestive tract other than the intestine, such as the esophagus [13] and stomach [14, 15]. 

In this study, we immunohistochemically examined the expressions of GPX1, CDX2, and Ki67 in 172 samples of human gastric adenocarcinomas. The association of the expression of GPX1 and CDX2 with various histopathological features was assessed, and the relationship between GPX1 and CDX2 was evaluated.

## 2. Material and Methods

### 2.1. Patient Data

Tumor specimens were obtained from 172 patients (108 males and 64 females; age range 24 to 82 years) who underwent surgery for gastric adenocarcinomas from November 2005 to April 2008. None of the patients had received prior chemotherapy or radiotherapy. All patients provided written informed consent. Clinical and pathological records and slides were available for all cases. HE-stained slides of gastric adenocarcinomas were reviewed. Histopathological examination indicated that 20 cases were well differentiated, 42 were moderately differentiated, and 110 were poorly differentiated. Among the 172 cases, 64 GAC samples were of intestinal type according to Lauren-type classification, 70 were of diffuse type, and 38 were of mixed type, respectively. According to the World Health Organization (WHO) histological classification, 142 patients were diagnosed as tubular type, 12 patients were diagnosed as mucinous type, 4 patients were diagnosed as of papillary type, and 14 patients were diagnosed as of signet ring cell type. A total of 35 cases had no lymph node involvement, 42 had one lymph node involved, and 95 had more than one lymph node involved. According to TNM classification, there were 10 cases at stageI, 18 at stage II, 80 at stage III, and 64 at stage IV.

### 2.2. Processing of Specimens and Immunohistochemistry

For protein expression analysis, 172 gastric adenocarcinomas and 16 adjacent normal mucosa (ANM) tissues samples were detected by using immunohistochemical staining. All of the tissue samples were histologically verified. Sections (4 *μ*m) of tissue blocks were transferred to an adhesive-coated slide. A 3-step immunoperoxidase technique using streptavidin-peroxidase (S-P) was employed for GPX1, CDX2, and Ki67 detection. All sections were routinely deparaffinized and rehydrated, and then the sections were rinsed in phosphate-buffered saline (PBS, pH = 7.4), and subsequently were treated for antigen retrieve (10 min, microwave oven, 10 mM EDTA, pH 8.0). After cooling at room temperature for 20 min, the sections were rinsed in PBS and immersed in 3% H_2_O_2_ for 15 min to block the endogenous enzymes. After being rinsed in PBS, the sections were incubated with normal goat serum at 37°C for 15 min to block nonspecific antibodies. The primary antibody was a polyclonal goat antiserum for GPX1 (Rabbit mAb, Eipt Mics, USA,1:50), CDX2 (Rabbit mAb, EPR2764Y, Maxim, China, ready for use), and Ki67 (Mouse mAb, MIB-1, Maxim, China, ready for use). After being incubated with primary antibody at 37°C for 60 min, the sections were rinsed in PBS, incubated with biotinylated secondary antibody (SP kit, Maxim, China), and rinsed in PBS again. After interaction with streptavidin-HRP (SP kit, Maxim, China) a rinse in PBS, the sections were visualized by reaction with 3,3′-diaminobenzidine and counterstained with hematoxylin. Normal gastric and intestinal mucosa were used as positive controls for GPX1 and CDX2, respectively. A lymph node was used as a positive control for Ki67, and normal goat serum and PBS substitution for the primary antibody were used as negative controls.

### 2.3. Scoring of the Results

All of the staining results were evaluated by two independent researchers (JH and HL) without the knowledge of the clinicopathological data. If the two investigators assigned different scores, consensus was obtained in all of the cases after discussion.

#### 2.3.1. GPX1

For the staining of GPX1, a positive stain was defined as brown stain observed in the cytoplasm. Tissues with no evidence of staining or only rare scattered positive cells less than 3% were recorded as negative. The immunohistochemical results were evaluated for the intensity and frequency of staining. The intensity of staining was graded as 0 (negative), 1 (weak), 2 (moderate), or 3 (strong). The frequency was graded from 0 to 4 by the percentage of positive cells as follows: grade 0, <3%; grade 1, 3–25%; grade 2, 25–50%; grade 3, 50–75%; and grade 4, more than 75%. The index score was the product of multiplication of the intensity and frequency grades, which was then classified using a 4-point scale: index score 0 = product of 0, index score 1 = products 1 and 2, index score 2 = products 3 and 4, and index score 3 = products 6 through 12 [16].

#### 2.3.2. CDX2

For the staining of CDX2 and Ki67, a positive stain was defined as brown stain observed in the nuclei. The whole area of slide was evaluated and positive CDX2 and positive Ki67 were defined by more than 10% positive cancer cells [17].

### 2.4. Statistical Analysis

All of the statistical analyses were performed with SPSS 8.0 software for Windows. The Kruskal-Wallis rank-sum test was used to compare GPX1 expression with clinicopathological factors, and the Wilcoxon rank-sum test was used to compare CDX2 expression with clinicopathological factors and both factors with Ki67. The survival analysis of patients was determined using the Kaplan-Meier method and Cox regression, and statistical evaluation was performed using the log rank test. *P* < .05 was considered statistically significant.

## 3. Results

### 3.1. Expression of GPX1 in GAC and Adjacent Normal Mucosa (ANM) Tissues

**Figure 1:**
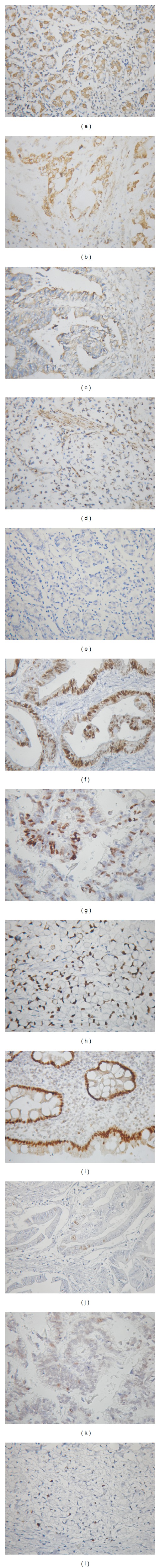
Immunohistochemical staining in GAC and ANM tissue. (a) GPX1 expression in ANM tissue (score 3). (b) GPX1 expression in well-differentiated GAC (score 3). (c) GPX1 expression in well-differentiated GAC (score 2). (d) GPX1 expression in signet-ring cell GAC (score 0). (e) CDX2 expression in ANM tissue (negative). ((f) and (g)) CDX2 expression in well-differentiated GAC (positive). (h) CDX2 expression in signet-ring cell GAC (negative). (i) CDX2 expression in normal colon mucosa (positive). ((j) and (k)) Ki67 expression in well-differentiated GAC (negative). (l) Ki67 expression in signet-ring cell GAC (negative). Original magnification: ×200.

Out of 172 gastric adenocarcinomas, 1 case (0.6%) had an index score of 3 (the case scored as a 3 was merged with those cases scored as a 2, statistically), 35 cases (20.3%) had index scores of 2, 96 cases (55.8%) had index scores of 1, and 40 cases (23.3%) had index scores of 0 (Figures 1(a)–1(d)). The total positive rate of GPX1 in all 172 cases of GAC was 76.7% (132/172). All 16 of the ANM tissues samples were scored as a 3. The expression of GPX1 in ANM tissues was significantly stronger than that in GAC (*Z* = −7.170, *P* = .000, *r* = .664). The higher positive rate of GPX1 was associated with better differentiation (*χ* ^2^ = 53.401, *P* = .000, *r* = .541). The total expressive rate of GPX1 was 100% (20/20) in the well-differentiated group, 92.9% (39/42) in the moderately differentiated group and 66.3% (73/110) in the poorly differentiated group. The positive rate of GPX1 was associated with Lauren type (*χ* ^2^ = 22.389, *P* = .000, *r* = .308). In 64 cases of intestinal type, the total positive rate was 92.2% (59/64), which was higher than that in the diffuse (61.4%, 43/70) and mixed types (78.9%, 30/38) (Table 1). The expression of GPX1 was associated with WHO type. The expression of GPX1 in the tubular type was stronger than that in the other types (*χ* ^2^ = 28.081, *P* = .000, *r* = .396). In 142 cases of tubular type, 36 cases were scored as 2, and 23 were scored as 1. In 30 cases of other types, no cases were scored as 2, and 13 were scored as 1. The expression of GPX1 was negatively associated with lymph node involvement (*χ* ^2^ = 9.710, *P* = .008, *r* = −.230). Out of 35 cases with no lymph node involvement, 12 cases were scored as 2, 19 were scored as 1, and 4 were scored as 0. Out of 42 cases with one lymph node involved, 10 cases were scored as 2, 25 were scored as 1, and 7 were scored as 0. Out of 95 cases with more than one lymph node involved, 14 cases were scored as 2, 52 were scored as 1, and 29 were scored as 0. The expression GPX1 was not associated with age, sex, or TNM stage (*P* > .05) (Table 1).

### 3.2. Expression of CDX2 in GAC and Adjacent Normal Mucosa (ANM) Tissues

Out of 172 gastric adenocarcinomas, 102 cases (59.3%) demonstrated positive expression of CDX2 and 70 cases (40.7%) were negative (Figures 1(e)–1(h)). Out of 16 ANM tissues samples, none was positive for CDX2. The positive rate for CDX2 in GAC was significantly higher than that in ANM tissues (*Z* = −4.540, *P* = .000, *r* = .332) (Figure 1). The higher positive rate of CDX2 was associated with better differentiation (*Z* = −5.336, *P* = .000, *r* = .398). The expressive rate of CDX2 was 95% in the well-differentiated group, 81.0% in the moderately differentiated group, and 44.5% in the poorly differentiated group. The positive rate of CDX2 was associated with Lauren type (*Z* = −3.962, *P* = .000, *r* = .291). In 64 cases of intestinal type, 52 cases were positive and the positive rate was 81.3%, which was higher than that in the diffuse and mixed types (50/108, 46.3%). The positive rate of CDX2 was negatively associated with lymph node involved (*Z* = −5.027, *P* = .000, *r* = −.335). In 34 cases of no lymph node involved, 29 cases (85.3%) were positive for CDX2. In 42 cases with one lymph node involved, 33 cases (78.6%) were positive. In 96 cases with more than one lymph node involvement, 40 cases (41.7%) were positive (Table 1). The positive rate of CDX2 was negatively associated with the TNM stage (*Z* = −2.243, *P* = .025, *r* = −.177). In 28 cases of stages I and II, 24 cases (85.7) were positive. In 144 cases of stage III and IV, 78 cases (54.2%) were positive. The expression CDX2 was not associated with age, sex, or WHO type (*P* > .05) (Table 1).

### 3.3. Prognostic Implication of GPX1 and CDX2 Expression in GAC

**Figure 2:**
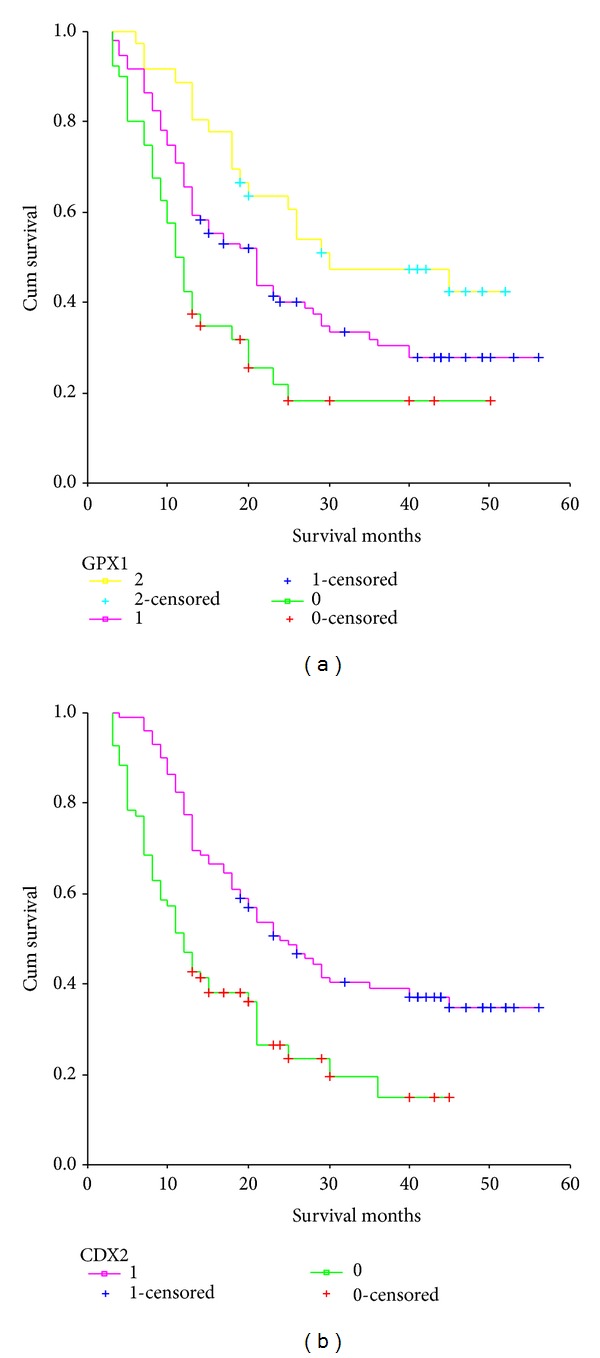
Survival curve of gastric cancer patients (a) GPX1 high expression group compared with GPX1 weak expression group and (b) CDX2 positive expression group compared with CDX2 negative expression group (*P* = .030 and .021).

The median survival time was 26 months for patients with GPX1 expression scored as 2, 17 months for patients scored as 1, and 11 months for patients scored as 0. The Kaplan-Meier method was used to analyze the association between the total survival rate and the expression of GPX1. The patients with stronger expression of GPX1 showed a significantly higher survival rate than patients with weaker expression of GPX1 (*P* = .030) (Figure 2). The median survival time of patients with positive CDX2 expression was 23 months, and that of the patients with negative CDX2 expression was 12 months. The Kaplan-Meier method was used to analyze the association between the total survival rate and the expression of CDX2. The patients with a higher positive rate of CDX2 showed a significantly higher survival rate than patients with a lower positive rate (*P* = .021) (Figure 2). Based on the Cox regression analysis of the 172 patients, CDX2 expression, lymph node metastasis, and TNM stage seemed to be independent prognostic indicators (*P* = .026, .011, and .001, resp., Table 2).

### 3.4. Correlation of GPX1 and CDX2 Expression with Ki67

The expression of GPX1 was negatively associated with Ki67. Cases of GPX1 scored as 2 had a higher Ki67 positive rate than those scored as 1 and 0 (*Z* = −3.843, *P* = .000, and *r* = −.294) (Figures 1(j)–1(l)). The positive rate of CDX2 was not associated with the positive rate of Ki67 (*Z* = −1.434, *P* = .152) (Table 3).

### 3.5. Correlation of GPX1 with CDX2 Expression in GAC

The expression of GPX1 was positively associated with CDX2 in GAC (*Z* = −4.754, *P* = .000, and *r* = .363, Table 4).

## 4. Discussion

Selenium has been shown to be effective in reducing carcinogenesis in animal model systems, and human studies supporting a protective role of this element have been reported. Selenoproteins whose ultimate levels can be influenced by selenium suggest a mechanism for how this element reduces cancer incidence. Normal levels of selenium that are present during homeostasis upregulate endogenous antioxidant defenses by increasing GPX activity [18]. GPX1 plays an important role in the protection of cells from oxidative stress by reducing hydrogen peroxide with glutathione. The GPX-1 gene has been found to be a frequent LOH locus in cancers of the lung, breast, and ovary [19]. In this study, we found a significant loss of GPX1 expression in GAC compared with ANM, which demonstrated that the loss of GPX1 was involved in the carcinogenesis of GAC. Reduced levels of GPX-1 may increase the risk or promote the development of cancer [20]. Similar results were found in this study. The loss of cytosolic expression of GPX1 was associated with poor differentiation and extensive lymph node involvement. Expression of GPX1 in the group of poorly differentiated carcinomas and the group with more than one lymph node involved was significantly lower than that in the groups with well or moderately differentiated carcinoma and no lymph node involvement. Moreover, stronger expression of GPX1 was associated with a favorable survival. The loss of GPX1 expression was associated with aggressiveness and poor outcome in patients with GAC. However, nuclear expression of GPX1 may have different effects. In hepatocellular carcinoma (HCC), selenium-binding protein 1 (SBP1) and GPX1 formed nuclear bodies and colocalized under oxidative stress. A decrease of SBP1 was linked with increased GPX1 activity, and it correlated with vascular invasion and a poor outcome [21]. GPX1 expression in different locations of tumor cells may have a different impact on tumor development, which warrants further investigation. GPX1 expression was associated with the Laurence type and WHO classification of tumor subtypes, but the mechanism is unclear. Accounting for the relationship of intestinal-type GAC with survival, the association of GPX1 and Lauren type may be incidental. GPX1 was found to be significantly increased and survivin was reduced following resveratrol treatment in non-small-cell lung carcinoma cells, which suggested that GPX1 might be involved in the inhibition of tumor cell proliferation [22]. In this study, stronger expression of GPX1 was found to be association with low Ki67 positive rate. This result suggested that the loss of GPX1 increased tumor cell proliferation.

CDX2 was found to be intensively involved in intestinal metaplastic differentiation [23]. CDX2 may stimulate intestinal proliferation and differentiation by transcriptional activation of intestine-specific proteins (MUC2, sucrase-isomaltase, and carbonic anhydraseI). Aberrant expression of CDX2 is prominent in intestinal-type gastric adenocarcinoma and CDX-2 may therefore play an important role in gastric carcinogenesis, especially in the intestinal type of GAC [24]. In this study, we found that CDX2 was not expressed in normal gastric mucosa, but it was expressed in 59.3% of GAC, which showed abnormal upregulation in GAC. Correa [25] proposed that human gastric carcinogenesis is a multistep process that progresses in the following order: chronic gastritis, atrophy, intestinal metaplasia, dysplasia, and gastric cancer. Although CDX2 was reported not to be associated with the intestinal metaplastic subtype [26], CDX2 was found to be intensively involved in intestinal metaplastic differentiation [23]. We demonstrated that CDX2 expression was associated with the Lauren type and WHO type of GAC in this study. The data demonstrated that CDX2 was expressed more in well differentiated, intestinal type and tubular type than that in the other types of GAC. These results suggest that CDX2 may play a role in the differentiation of the tumor cells after carcinogenesis. In this study, CDX2 expression was decreased more in the group with more than one lymph node involved and in the stage III/IV (TNM) group than in the groups with no lymph node involvement/one lymph node involved and the stage I/II (TNM) group. We also found that CDX2 expression was associated with a favourable outcome. Similarly, CDX2 was reported as a prognostic factor that acted as a marker of good outcome in patients with gastric cancer [27]. Based on our results, CDX2 transcriptional factor might act as a tumour suppressor [28, 29].

In our study, we demonstrated that the expression of GPX1 was correlated positively with the expression of CDX2 in GAC, but the mechanism is not clear. 

GPX is involved in resistance to Adriamycin [30]. Moreover, CDX2 activates the *Multidrug Resistance 1* gene in gastrointestinal cancers [31]. We speculate that GPX1 and CDX2 either cooperate or simply coexist in their roles in drug resistance in GAC, which warrants further investigation.

## Figures and Tables

**Table 1:** Relationship of GPX1 and CDX2 expression with clinicopathological factors of GAC.

Factors	*n*	GPX1 score			CDX2	
		0	1	2	−	+
Age						
<50	59	17	35	7	26	33
≥50	113	23	61	29	44	69
Sex						
Male	108	28	55	25	39	69
Female	64	12	41	11	31	33
Differentiation						
Well	20		5	15*	1	19*
Moderate	42	3	25	14	8	34
Poor	110	37	66	7	61	49
Lauren type						
Intestinal	64	5	31	28*	12	52*
Diffuse	70	27	40	3	38	32
Mixed	38	8	25	5	20	18
WHO type						
Tubular	142	23	83	36*	54	88
Mucinous	12	5	7		6	6
Papillary	4		4			4
Signet-ring cell	14	12	2		10	4
Lymph node involvement (number)						
No	35	4	19	12*	6	29*
One	42	7	25	10	9	33
>1	95	29	52	14	55	40
TNM stage						
I	10	1	7	2	3	7*
II	18		10	8	1	17
III	80	20	46	14	36	44
IV	64	19	33	12	30	34

*P < .05.

**Table 2:** Association of various factors with overall survival by logistic regression.

Variable	*B*	S.E.	Wald	df	Sig	*R*	Exp(*B*)
Age	.2276	.2233	1.0388	1	.3081	.0000	1.2556
Sex	−.1806	.2182	.6848	1	.4079	.0000	.8348
Differentiation	.0216	.2534	.0073	1	.9321	.0000	1.0218
Lauren type	−.2320	.1691	1.8834	1	.1700	.0000	.7929
WHO type	.0235	.1917	.0150	1	.9026	.0000	1.0237
LN involved	.2983	.1154	6.6818	1	.0097	.0662	1.3475
TNM stage	.3888	.1525	6.4967	1	.0108	.0649	1.4752
Ki67	.4773	.4485	1.1323	1	.2873	.0000	1.6117
GPX1	−.3468	.1837	3.5629	1	.0591	−.0383	.7069
CDX2	−.5168	.2321	4.9561	1	.0260	−.0526	.5964

**Table 3:** Association of GPX1 and CDX2 with Ki67 in GAC.

	*n*	GPX1 score		CDX2	
		0-1	2	−	+
Ki67					
−	47	28	19*	15	32
**+**	125	108	17	55	70

*P = .000.

**Table 4:** Association of GPX1 with CDX2.

	GPX1 score		
	0	1	2
CDX2			
−	28	36	6
**+**	12	60	30

*P* = .000.

## References

[1] Lambert R, Guilloux A, Oshima A (2002). Incidence and mortality from stomach cancer in Japan, Slovenia and the USA. *International Journal of Cancer*.

[2] Ferlay J, Steliarova-Foucher E, Lortet-Tieulent J (2013). Cancer incidence and mortality patterns in Europe: estimates for 40 countries in 2012. *European Journal of Cancer*.

[3] Chen W, Zheng R, Zhang S (2013). Report of incidence and mortality in China cancer registries, 2009. *Chinese Journal of Cancer Research*.

[4] Siewert JR, Böttcher K, Stein HJ, Roder JD (1998). Relevant prognostic factors in gastric cancer: ten-year results of the German Gastric Cancer Study. *Annals of Surgery*.

[5] Laimer K, Fong D, Gastl G (2008). EpCAM expression in squamous cell carcinoma of the oral cavity: frequency and relationship to clinicopathologic features. *Oral Oncology*.

[6] Halliwell B (1999). Antioxidant defence mechanisms: from the beginning to the end (of the beginning). *Free Radical Research*.

[7] Miyamoto Y, Koh YH, Park YS (2003). Oxidative stress caused by inactivation of glutathione peroxidase and adaptive responses. *Biological Chemistry*.

[8] Ray G, Husain SA (2002). Oxidants, antioxidants and carcinogenesis. *Indian Journal of Experimental Biology*.

[9] Brigelius-Flohé R, Kipp A (2009). Glutathione peroxidases in different stages of carcinogenesis. *Biochimica et Biophysica Acta*.

[10] Min SY, Kim HS, Jung EJ (2012). Prognostic significance of glutathione peroxidase 1 (GPX1) down-regulation and correlation with aberrant promoter methylation in human gastric cancer. *Anticancer Research*.

[11] Gross I, Duluc I, Benameur T (2008). The intestine-specific homeobox gene Cdx2 decreases mobility and antagonizes dissemination of colon cancer cells. *Oncogene*.

[12] Bonhomme C, Duluc I, Martin E (2003). The Cdx2 homeobox gene has a tumour suppressor function in the distal colon in addition to a homeotic role during gut development. *Gut*.

[13] Eda A, Osawa H, Satoh K (2003). Aberrant expression of CDX2 in Barrett’s epithelium and inflammatory esophageal mucosa. *Journal of Gastroenterology*.

[14] Almeida R, Silva E, Santos-Silva F (2003). Expression of intestine-specific transcription factors, CDX1 and CDX2, in intestinal metaplasia and gastric carcinomas. *Journal of Pathology*.

[15] Barros R, Camilo V, Pereira B, Freund J-N, David L, Almeida R (2010). Pathophysiology of intestinal metaplasia of the stomach: emphasis on CDX2 regulation. *Biochemical Society Transactions*.

[16] Peng DF, Razvi M, Chen H (2009). DNA hypermethylation regulates the expression of members of the Mu-class glutathione S-transferases and glutathione peroxidases in Barrett’s adenocarcinoma. *Gut*.

[17] Mizoshita T, Tsukamoto T, Nakanishi H (2003). Expression of Cdx2 and the phenotype of advanced gastric cancers: relationship with prognosis. *Journal of Cancer Research and Clinical Oncology*.

[18] Gan L, Liu Q, Xu H-B, Zhu Y-S, Yang X-L (2002). Effects of selenium overexposure on glutathione peroxidase and thioredoxin reductase gene expressions and activities. *Biological Trace Element Research*.

[19] Martinez A, Walker RA, Shaw JA, Dearing SJ, Maher ER, Latif F (2001). Chromosome 3p allele loss in early invasive breast cancer: detailed mapping and association with clinicopathological features. *Journal of Clinical Pathology*.

[20] Hu YJ, Diamond AM (2003). Role of glutathione peroxidase 1 in breast cancer: loss of heterozygosity and allelic differences in the response to selenium. *Cancer Research*.

[21] Huang C, Ding G, Gu C (2012). Decreased selenium-binding protein 1 enhances glutathione peroxidase 1 activity and downregulates HIF-1*α* to promote hepatocellular carcinoma invasiveness. *Clinical Cancer Research*.

[22] Hu Y, Rahlfs S, Mersch-Sundermann V, Becker K (2007). Resveratrol modulates mRNA transcripts of genes related to redox metabolism and cell proliferation in non-small-cell lung carcinoma cells. *Biological Chemistry*.

[23] Mizoshita T, Inada K-I, Tsukamoto T (2001). Expression of Cdx1 and Cdx2 mRNAs and relevance of this expression to differentiation in human gastrointestinal mucosa—with special emphasis on participation in intestinal metaplasia of the human stomach. *Gastric Cancer*.

[24] Seno H, Oshima M, Taniguchi M-A (2002). CDX2 expression in the stomach with intestinal metaplasia and intestinal-type cancer: prognostic implications. *International Journal of Oncology*.

[25] Correa P (1992). Human gastric carcinogenesis: a multistep and multifactorial process—first American Cancer Society Award lecture on cancer epidemiology and prevention. *Cancer Research*.

[26] Lee BH, Kim N, Lee HS (2012). The role of CDX2 in intestinal metaplasia evaluated using immunohistochemistry. *Gut and Liver*.

[27] Wang XT, Wei WY, Kong FB (2012). Prognostic significance of Cdx2 immunohistochemical expression in gastric cancer: a meta-analysis of published literatures. *Journal of Experimental & Clinical Cancer Research*.

[28] Hayes S, Ahmed S, Clark P (2011). Immunohistochemical assessment for Cdx2 expression in the Barrett metaplasia-dysplasia-adenocarcinoma sequence. *Journal of Clinical Pathology*.

[29] Park DY, Srivastava A, Kim GH (2010). CDX2 expression in the intestinal-type gastric epithelial neoplasia: frequency and significance. *Modern Pathology*.

[30] Lee W-P, Lee C-L, Lin H-C (1996). Glutathione S-transferase and glutathione peroxidase are essential in the early stage of Adriamycin resistance before P-glycoprotein overexpression in HOB1 lymphoma cells. *Cancer Chemotherapy and Pharmacology*.

[31] Takakura Y, Hinoi T, Oue N (2010). CDX2 regulates multidrug resistance 1 gene expression in malignant intestinal epithelium. *Cancer Research*.

